# Using the Generalized Index of Dissimilarity to Detect Gene-Gene Interactions in Multi-Class Phenotypes

**DOI:** 10.1371/journal.pone.0158668

**Published:** 2016-08-24

**Authors:** Jaeyong Yee, Yongkang Kim, Taesung Park, Mira Park

**Affiliations:** 1 Department of Physiology and Biophysics, Eulji University, Daejeon, Korea; 2 Department of Statistics, Seoul National University, Seoul, Korea; 3 Department of Preventive Medicine, Eulji University, Daejeon, Korea; Case Western Reserve University, UNITED STATES

## Abstract

To find genetic association between complex diseases and phenotypic traits, one important procedure is conducting a joint analysis. Multifactor dimensionality reduction (MDR) is an efficient method of examining the interactions between genes in genetic association studies. It commonly assumes a dichotomous classification of the binary phenotypes. Its usual approach to determining the genomic association is to construct a confusion matrix to estimate a classification error, where a binary risk status is determined and assigned to each genotypic multifactor class. While multi-class phenotypes are commonly observed, the current MDR approach does not handle these phenotypes appropriately because the thresholds for the risk statuses may not be clear. In this study, we suggest a new method for estimating gene-gene interactions for multi-class phenotypes. Our approach adopts the index of dissimilarity (IDS) as an evaluation measure. This is analytically equivalent to the common association measure of balanced accuracy (BA) for the binary traits, while it is not required to determine the risk status for the estimation. Moreover, it is easily expandable to the generalized index of dissimilarity (GIDS), which has an explicit form that can handle any number of categories. The performance of the proposed method was compared with those of other approaches via simulation studies in which fifteen genetic models were generated with three class outcomes. A consistently better performance was observed using the proposed method. The effect of a varying number of categories was examined. The proposed method was also illustrated using real genome-wide association studies (GWAS) data from the Korean Association Resource (KARE) project.

## Introduction

Recently, genome-wide association studies (GWAS) have been popular for finding the association between a single nucleotide polymorphism (SNP) and complex traits. The traditional analysis tested a single SNP at a time and selected top few SNPs. However, this single-locus approach has evolved into a multiple-loci approach because most complex diseases are associated with multiple genes and their interactions [1–3].

Several statistical methods can be used for detection of gene-gene interactions. The multifactor dimensionality reduction (MDR) method is a non-parametric approach that can efficiently identify higher-order interactions between genes [4,5]. Originally, MDR was intended for analysis of genomic datasets with binary outcomes (e.g., case-control or affected-unaffected). It reduces the dimension of the multi-locus genomic factors by considering all possible combinations in a one-dimensional array, each element of which is classified as either high-risk or low-risk [6]. Thus a 2×2 confusion matrix is used to estimate the prediction error or, equivalently, the classification accuracy. Through the advent of a number of alternative measures [7–9], the confusion matrix scheme has remained.

MDR has been expanded to be applicable to various types of phenotypic traits. For quantitative traits, generalized MDR (GMDR) accomplishes this by covariate adjustment [10], while quantitative MDR (QMDR) compares the mean value of each multi-locus genotype to the overall mean [11]. A common strategy may be to assess the trait values within each genotypic multifactor class by assigning a binary risk status to each one. Test accuracy may be used or be replaced by another statistic such as the T-statistic. Multivariate versions of the GMDR and QMDR have also inherited the binary risk status scheme [12–14]. For ordinal categorical phenotypes, ordinal MDR (OMDR) was proposed [15]. It estimates a series of odds ratios for each multifactor class to set the OMDR classifier. Kendall’s tau-b is the statistic of choice for the association measure. It keeps the frame of the confusion matrix because the OMDR classifiers represent an extension of binary case-control and high-low risks. For example, a subject observed to be in a normal, pre-, mild-, or severe-obese class may be found to be in one of the four risk statuses depending on the multifactor class it is in.

Some phenotypes may consist of multiple categories which are not ordinal. For example, a combination of systolic and diastolic blood pressure is used as an indicator for hypertension, and the classification for the two combined is more complex than a simple ordinal in that its classification depends on ‘AND’ and ‘OR’ logics [16]. However, no MDR extension applicable to traits with multiple categories has been proposed until recently. Furthermore, all of the earlier MDR extensions need a procedure for assigning the risk status, which requires clear criteria for the thresholds for the risk statuses.

Our aim in this paper is to propose a new MDR extension to estimate the genomic association of a gene-gene interaction with a multi-class phenotype. Our approach uses a measure that may be utilized for a multi-class phenotype without the need to explicitly construct a confusion matrix. We first introduce the index of dissimilarity (IDS) and the generalized IDS (GIDS) measures [17]. We then suggest a way to find gene-gene interactions for a composite multi-class trait using these measures. This may be considered a dimensional reduction in both the genotype (multi-locus) and phenotype (multi-class) parts. We conducted two types of simulations to investigate the performance of the proposed method. We analyzed a real GWAS data from the Korean Association Resource (KARE) project to illustrate the method [18].

## Methods

### Definition of IDS and GIDS

The index of dissimilarity (IDS) was originally conceived as a measure of segregation to quantify the extent of uneven distribution of two population groups in several areas of a city, such as census tract [17]. It is defined as follows when the number of areas to consider is *I*.

$$IDS=\frac{1}{2}\sum_{i=1}^{I}\left|\frac{n_{i1}}{n_{•1}}-\frac{n_{i2}}{n_{•2}}\right|\;\text{where}\;n_{•j}=\sum_{i=1}^{I}n_{ij}$$ (1)

Because *n*_*ij*_ represents the number of subjects in the *i*^*th*^ area and *j*^*th*^ group, IDS can be interpreted as the proportion in one group or the other that must be moved in order to achieve an even distribution [17]. A generalized index of dissimilarity (GIDS) is an extension of the IDS to an arbitrary number of population groups. It is defined as follows.

$$GIDS=\frac{1}{2}\frac{\sum_{j=1}^{J}\sum_{i}\left|n_{ij}-E_{ij}\right|}{\sum_{j=1}^{J}n\cdot P_{•j}\cdot \left(1-P_{•j}\right)}\;\text{where}\;n=\sum_{j=1}^{J}n_{•j},\;E_{ij}=\frac{n_{i•}\cdot n_{•j}}{n},P_{•j}=\frac{n_{•j}}{n}$$ (2)

Note that the denominator in this equation defines the maximum possible value of the corresponding numerator, so that GIDS would have values between 0 and 1. When *J* = 2, the GIDS is reduced to the IDS as shown below.

$$\begin{array}{l} GIDS(J=2)=\frac{1}{2}\frac{\sum_{i}\left|n_{i1}-\frac{\left(n_{i1}+n_{i2}\right)n_{•1}}{n}\right|+\sum_{i}\left|n_{i2}-\frac{\left(n_{i1}+n_{i2}\right)n_{•2}}{n}\right|}{n_{•1}\left(1-\frac{n_{•1}}{n}\right)+n_{•2}\left(1-\frac{n_{•2}}{n}\right)}\\ \;=\frac{1}{2}\frac{\sum_{i}\left|n_{i1}n_{•2}-n_{i2}n_{•1}\right|+\sum_{i}\left|-\left(n_{i1}n_{•2}-n_{i2}n_{•1}\right)\right|}{2n_{•1}n_{•2}}\\ \;=\frac{1}{2}\sum_{i}\left|\frac{n_{i1}}{n_{•1}}-\frac{n_{i2}}{n_{•2}}\right|\\ \;=IDS \end{array}$$ (3)

### Finding gene-gene interaction using GIDS

**Fig 1:**
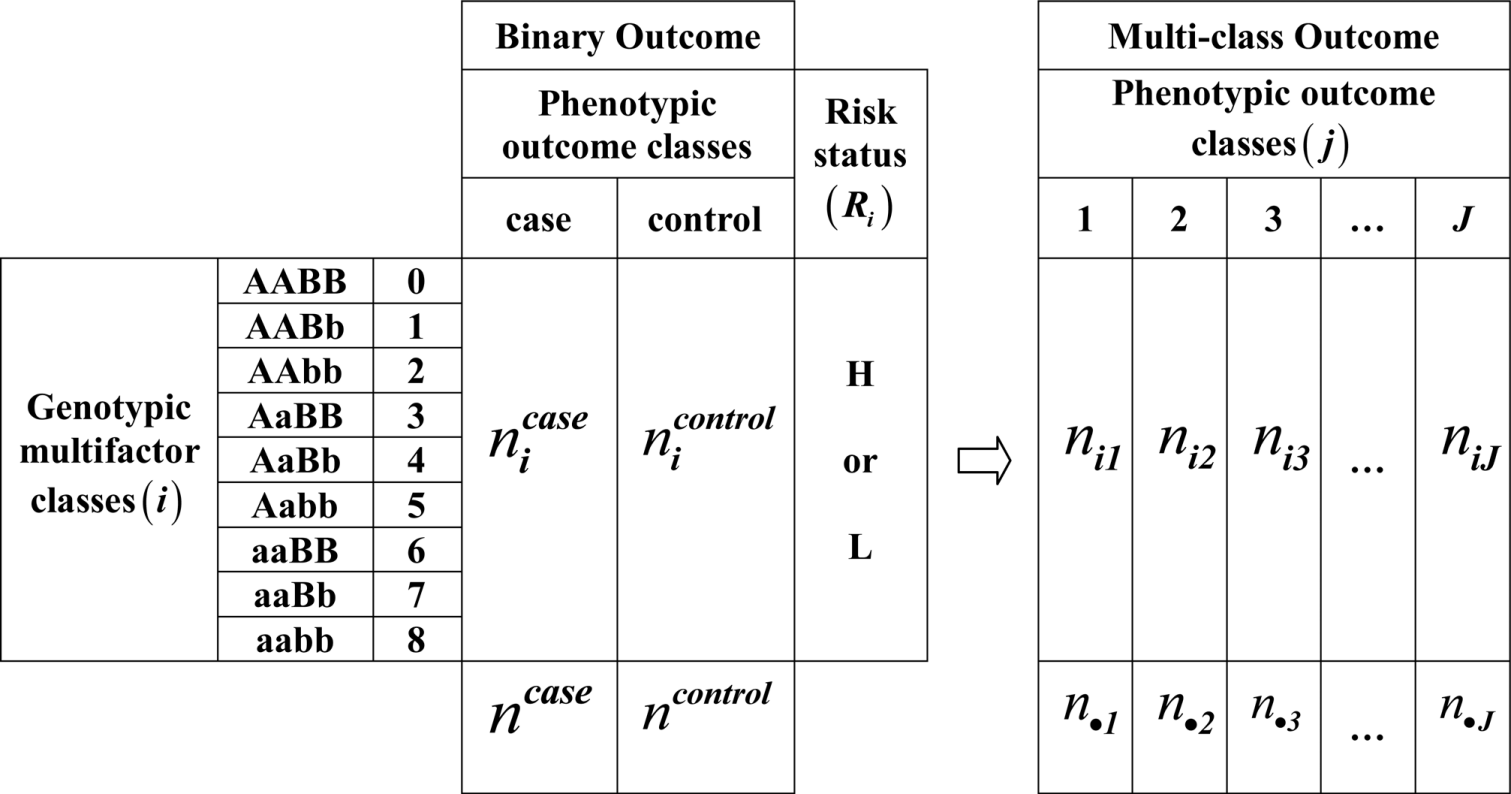
Schematics for the association measure for 2-order interaction with binary and multiple outcome classes. With binary outcome, phenotype is observed as either case or control for each sample, sorted by the multifactor class, and cumulated separately for case and control. Risk status (*R*_*i*_) for each multifactor class is classified as high (H) or low (L) by comparing the ratio *n*_*i*_^*case*^/*n*_*i*_^*control*^ with a certain threshold. With multi-class outcome, only the sorted and cumulated numbers of samples (*n*_*ij*_) for each multifactor class are required, where *i* and *j* represent the genotypic and phenotypic class, respectively.

In terms of genomic data, areas may be replaced by genotypic classes, while groups can be understood as the phenotypic outcome classes. The data structure with more than two outcome classes is depicted on the right side of Fig 1. Subject with indices *i* and *j* should have an *i*^*th*^ multi-locus genotype with a *j*^*th*^ multi-class phenotype. Therefore, the numerator of the GIDS in Eq (2) sums the extent of observed uneven distributions by each outcome class, while the denominator estimates the sum of the maximal possible unevenness in each outcome class. Procedures to obtain the SNP combinations with significant gene-gene interactions can be summarized as follows.

[Step 1] Construction of a 2-way table

At the first stage, we constructed a 2-way contingency table of the genotypes and the disease status. For *k*-locus interactions with *J* phenotype categories, a 3^*k*^×*J* contingency table is constructed for each SNP combination. For example, when *k* = 3, the element of the table should be *n*_*ij*_ as depicted in Fig 1.

[Step 2] Calculation of GIDS

Now that *n*_*ij*_ is defined, the GIDS can be obtained using the Eq (2). The SNP combination that has a larger GIDS value is regarded as having a stronger genomic association.

[Step 3] Estimation of p-values

First, randomly permute the whole dataset in order to make the resultant dataset consistent with the null hypothesis. Then apply the previous step to identify the maximum GIDS value for the permuted dataset. Repeat the permutation and calculation for the maximum GIDS. Collection of the maximum GIDS values, obtained from each iteration, should make the null distribution that can be used to estimate the p-values for the GIDS values from the original dataset. In that way, we can compare models with different orders of interactions and identify the best model based on these p-values [19–21].

### Equivalence of balanced accuracy (BA) and IDS

To estimate the genomic association in MDR, balanced accuracy (BA) utilizes the concept of risk status as depicted on the left side of Fig 1. Each sample that falls into a particular genotypic multifactor class is classified according to criteria set up by the combined condition of the outcome class and the risk status. A confusion matrix is constructed with the classification as follows.

$$TP=\sum_{i}^{R_{i}=H}n_{i}^{case},\;TN=\sum_{i}^{R_{i}=L}n_{i}^{control},\;FN=\sum_{i}^{R_{i}=L}n_{i}^{case},\;FP=\sum_{i}^{R_{i}=H}n_{i}^{control}$$ (4)

Positive (P) or negative (N) classifications are determined by outcome classes corresponding to ‘case’ or ‘control’. As shown in Eq (4), risk statuses of high (H) and low (L) matched with ‘case’ and ‘control’, respectively, are tagged as true (T), while a mismatch is tagged as false (F). Risk status is commonly defined as below.

$$R_{i}=[\begin{array}{c} H\;;n_{i}^{case}/n_{i}^{control}\geq n^{case}/n^{control}\\ L\;;n_{i}^{case}/n_{i}^{control}<n^{case}/n^{control} \end{array}$$ (5)

BA is then defined within the above schematics.

$$BA=\frac{1}{2}\left(\frac{TP}{TP+FN}+\frac{TN}{TN+FP}\right)$$ (6)

The IDS may be applied to data with case and control trait groups without considering the risk status, as below.

$$IDS=\frac{1}{2}\sum_{i}\left|\left(\frac{n_{i}^{case}}{n^{case}}\right)-\left(\frac{n_{i}^{control}}{n^{control}}\right)\right|$$ (7)

Using Eqs (4) and (5), along with the identities of *n*^*case*^ = *TP* + *FN* and *n*^*control*^ = *TN* + *FP*, Eq (7) can be rearranged as follows.

$$\begin{array}{l} IDS=\frac{1}{2}\left(\sum_{\frac{n_{i}^{case}}{n^{case}}\geq \frac{n_{i}^{control}}{n^{control}}}\left(\frac{n_{i}^{case}}{n^{case}}-\frac{n_{i}^{control}}{n^{control}}\right)+\sum_{\frac{n_{i}^{case}}{n^{case}}<\frac{n_{i}^{control}}{n^{control}}}\left(\frac{n_{i}^{control}}{n^{control}}-\frac{n_{i}^{case}}{n^{case}}\right)\right)\\ \;=\frac{1}{2}\left(\frac{TP}{n^{case}}-\frac{FP}{n^{control}}+\frac{TN}{n^{control}}-\frac{FN}{n^{case}}\right)\\ \;=\left(\frac{TP}{n^{case}}+\frac{TN}{n^{control}}\right)-1\\ \;=2\cdot BA-1 \end{array}$$ (8)

The IDS and BA are now shown to be analytically equivalent except in the spanning range. Knowing that the possible range of BA is from 0.5 to 1, IDS spans the values from 0 to 1: absolutely no association corresponds to 0.5 for BA and 0 for IDS, while a maximum association gives rise to a value of 1 for both BA and the IDS.

### Consequence of making the explicit use of the risk status unnecessary

Estimation of genomic association for data with more than two outcome classes, as depicted on the right side of Fig 1, would make it difficult to derive BA because it would require setting multiple thresholds between adjacent outcome classes for determining a risk status. The GIDS can take advantage of a definition that does not require setting the threshold. Assigning ‘true’ or ‘false’ by comparing the risk status with the outcome class then becomes an unnecessary step. An important consequence of this is that there is no restriction on the number of outcome classes that can be analyzed with. When there are more than two outcome classes, the thresholds that should be set may also become less obvious. The GIDS does not require assigning the risk status to each multi-locus genotype, making it unnecessary to set the thresholds for the discrimination among the risk statuses as well. For example, if there are three possible outcome classes, another risk status in addition to H and L should be defined along with the appropriate thresholds. Eliminating the need for this decision enables extending the number of outcome classes that can be analyzed. Consequently, the natural extension of the IDS to GIDS may be devised without any ambiguity. The GIDS may then measure the genomic association strength between the genotypic multifactor classes and the multi-class phenotypes.

## Results

We conducted two simulation studies and analysed a dataset from a real genome-wide association study. For simulation I, a genetic dataset with a categorical trait was generated. For simulation II, a situation was considered in which the categorical trait was derived from the classification of the continuous variable. Data from the Korean Association Resource (KARE) project [18] was analysed as the real example. Detailed schemes and results are as follows.

### Simulation study I

**Fig 2:**
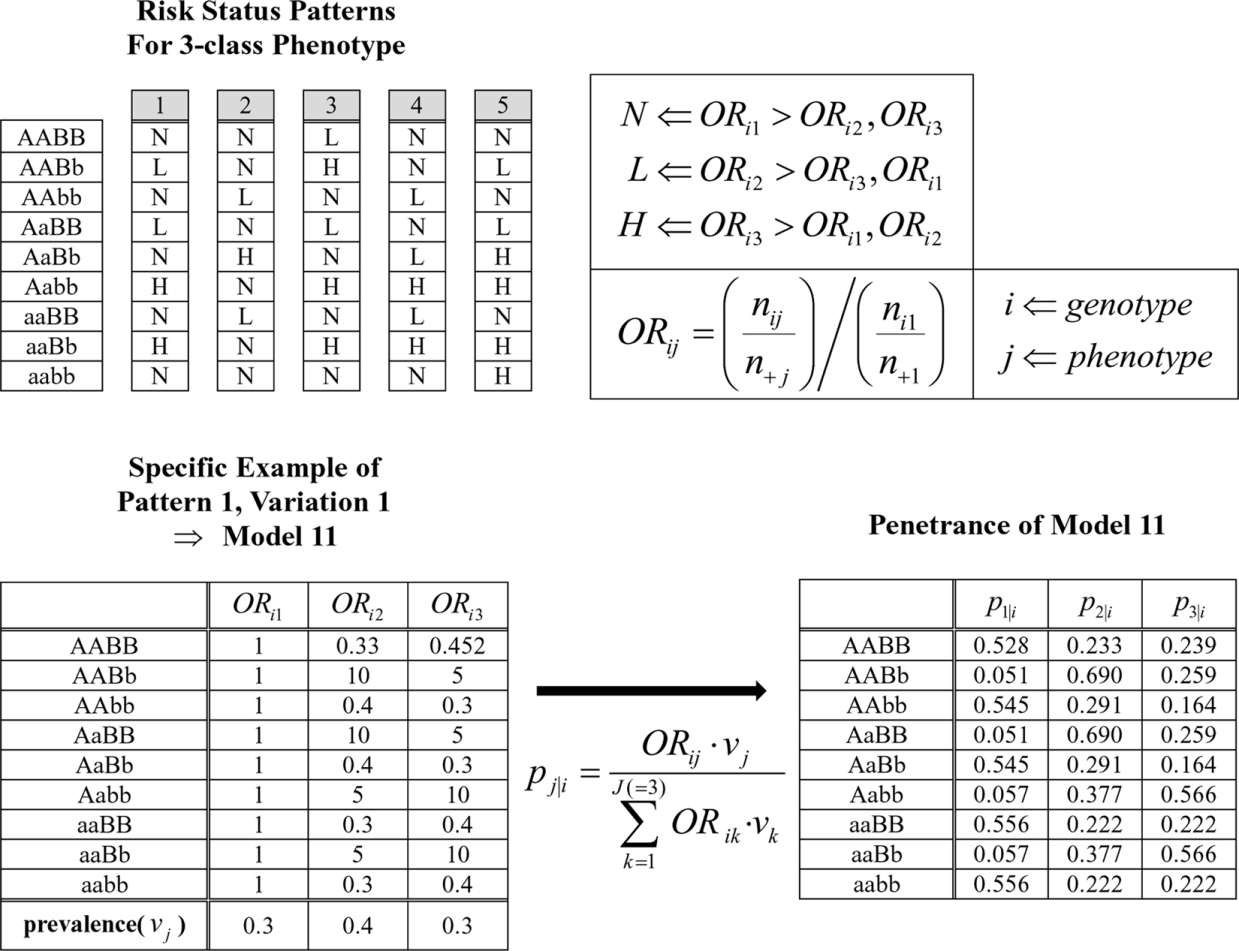
Penetrance assigning scheme in simulation I. Five risk status patterns for 3-class phenotype were devised in [15]. Pattern tells which of the 3-classes has the largest odds ratio. One of the specific set of the odds ratios that satisfies the pattern 1 is shown here. With fixed prevalence for each class, a set of penetrance can be determined.

A genetic dataset with a categorical trait of three classes was generated to demonstrate the ability of the GIDS to correctly identify the causal pair. A two-way interaction between single nucleotide polymorphisms (SNPs) was considered with a single causal pair intended. As depicted in Fig 2, five different risk status patterns were developed in [15]. One of the three classes of the trait would be related to the largest odds ratio according to the pattern over nine multi-locus genotypes. While there were many specific sets of odds ratios that could satisfy each pattern, three variations for each pattern were modelled as in the S1 Table of reference [15]. In Fig 2, actual odds ratios for the first variation of the first pattern are shown. Prevalence of each of the three phenotypic classes, *v*_*j*_, was fixed as 0.3, 0.4, and 0.3, throughout the models. With those prevalence and odds ratios, the set of penetrance, *p*_*j|i*_, should be determined. Then the penetrance was used as the probability of assigning the phenotypic class to each simulated sample. Minor allele frequencies (MAFs) were set as 0.5 for the first two patterns, and 0.3 for the rest of them. In all, 5×3 = 15 models were considered. For each model, 100 simulated data files were generated, with a sample size of 400, and 20 SNPs.

**Fig 3:**
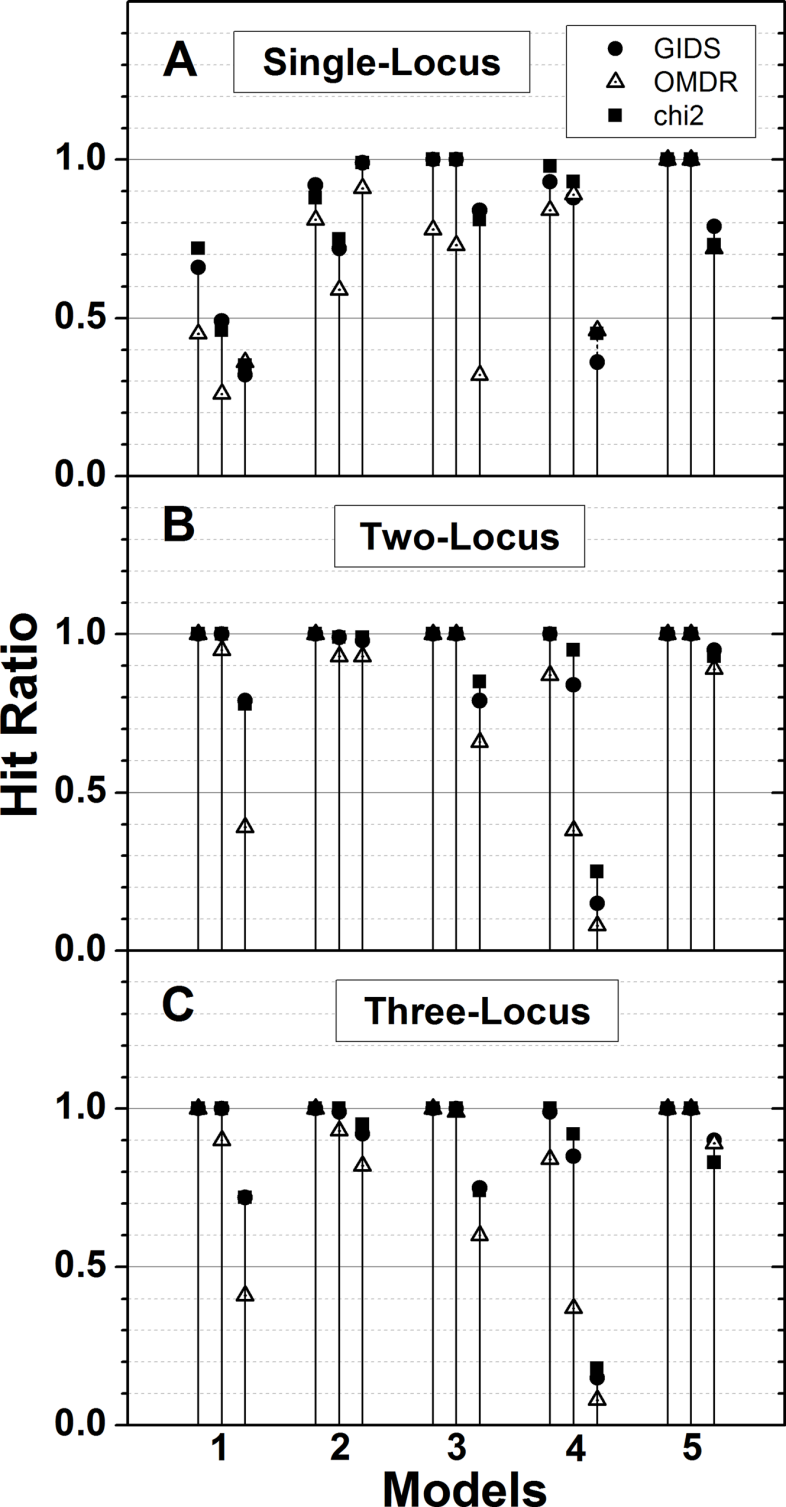
Comparison of performance (simulation dataset I). Performances of GIDS and OMDR as well as *χ*^2^ are compared for the simulation datasets of fifteen models grouped in five patterns. All models are designed to have a single causal pair with two-way interactions and three phenotypic classes. Hit ratio is defined as the ratio identifying the causal pair correctly in “Two-Locus”. In “Single-Locus” it is the ratio identifying either of the causal pairs. The ratio identifying the combination that includes the causal pair is plotted in “Three-Locus”.

In Fig 3, the performance on the simulation dataset I is plotted. It should be noted that simulation dataset I is based on five two-way interaction patterns with three sets of penetrance variations within each pattern. It should also be noted that the number of the categorical phenotypic class in this dataset is three instead of the usual two, which is the reason that the GIDS was introduced. Performance was examined by the hit ratio, which is the ratio of the correct identification of a causal pair. Three sets of hit ratios estimated by the GIDS and OMDR [15] as well as χ^2^ are compared in Fig 3. In addition to the two-locus classifier, which indicates the intended two-way interactions, one- and three-locus classifiers were also examined. Single-locus hit ratios were defined as the identification rate of either of the causal pair. Using the three-locus classifier, the identification rate of the three SNP combinations, two out of which are the causal pair, was defined as the hit ratio. In this way, the ability of the GIDS to choose the true order of interaction over adjacent orders may be examined. The GIDS consistently showed a better performance than the OMDR for the most of the cases plotted. Average hit ratios for the two-locus classifier were 0.90 and 0.81 for the GIDS and OMDR, respectively. For every model examined, the hit ratio of the GIDS was higher than that of the OMDR, as can be seen in Fig 3B. For single- and three-locus, the plots show similar trends, except in the performance inversion between the GIDS and OMDR in three out of fifteen models in Fig 3A. The average hit ratios are 0.79 and 0.67 for single-locus and 0.88 and 0.79 for three-locus, respectively, with the GIDS and OMDR. These values are expectedly lower than those in the two-locus model, which is the identification of the true causal pair. Performances by GIDS and *χ*^2^ are essentially indistinguishable except with model 33, 42 and 43. Excluding them, overall hit ratios for the three kinds of locus classifiers were estimated to be 0.81, 0.96, and 0.94, respectively, for both GIDS and *χ*^2^. Here model 43 means the third variation within the pattern 4, plotted as the third one of the three grouped as model 4. Including them, the performance of *χ*^2^ was about 2% higher than that of GIDS. Only these three, out of all 15 models, turned out not to have cells with penetrance smaller than 10%. *χ*^2^ seems to take more advantage of the lack of extreme cell counts than GIDS. It can be concluded, with simulation I, that GIDS is able to estimate correct genomic association for multi-class phenotypes while showing the best performance for the order of the true causal interaction, which is 2-order with this simulation data.

### Simulation study II

Quantitative phenotype was generated in simulation II. Now the penetrance table dictated the choice of the distribution function according to Eq (9), yielding the phenotype value.

$$y|\left(SNP_{1}=i,SNP_{2}=j\right)\sim [\begin{array}{l} N\left(f_{ij},\sigma_{L}^{2}\right)\;,\;if\;f_{ij}<\bar{f}\\ \left(N\left(f_{ij}-\alpha ,\sigma_{H}^{2}\right)+N\left(f_{ij}+\alpha ,\sigma_{H}^{2}\right)\right)/2\;,\;if\;f_{ij}\geq \bar{f} \end{array}$$ (9)

Here *y* represents the phenotype value associated with two interacting SNPs, while *f*_*ij*_ and $\bar{f}$ denote the penetrance and the average of them, respectively. Both *i* and *j* have the values of 0, 1, 2 or AA, Aa, aa, making 9 distinct values for *ij*. With a single causal pair intended, penetrance table was taken from the models in [22]. According to the result of a comparison between *f*_*ij*_ and $\bar{f}$, the phenotype value was generated from either of the distribution as given in Eq (9). Variances of σ_*L*_ and σ_*H*_ in the distribution function were chosen independently out of 0.8, 1.0, and 1.2 such that 9 combinations could be formed. The parameter α was chosen as 0.4 so that the normal distributions could overlap adequately. There were total of 70 penetrance models [22], which were grouped into seven different heritability values of 0.01, 0.025, 0.05, 0.1, 0.2, 0.3, and 0.4. Two different MAFs of 0.2 and 0.4 were used. Overall, 70×9 = 630 different conditions were set up, 90 for each heritability value. For each condition, 100 simulated data files were generated with a sample size of 400 and 20 SNPs.

**Fig 4:**
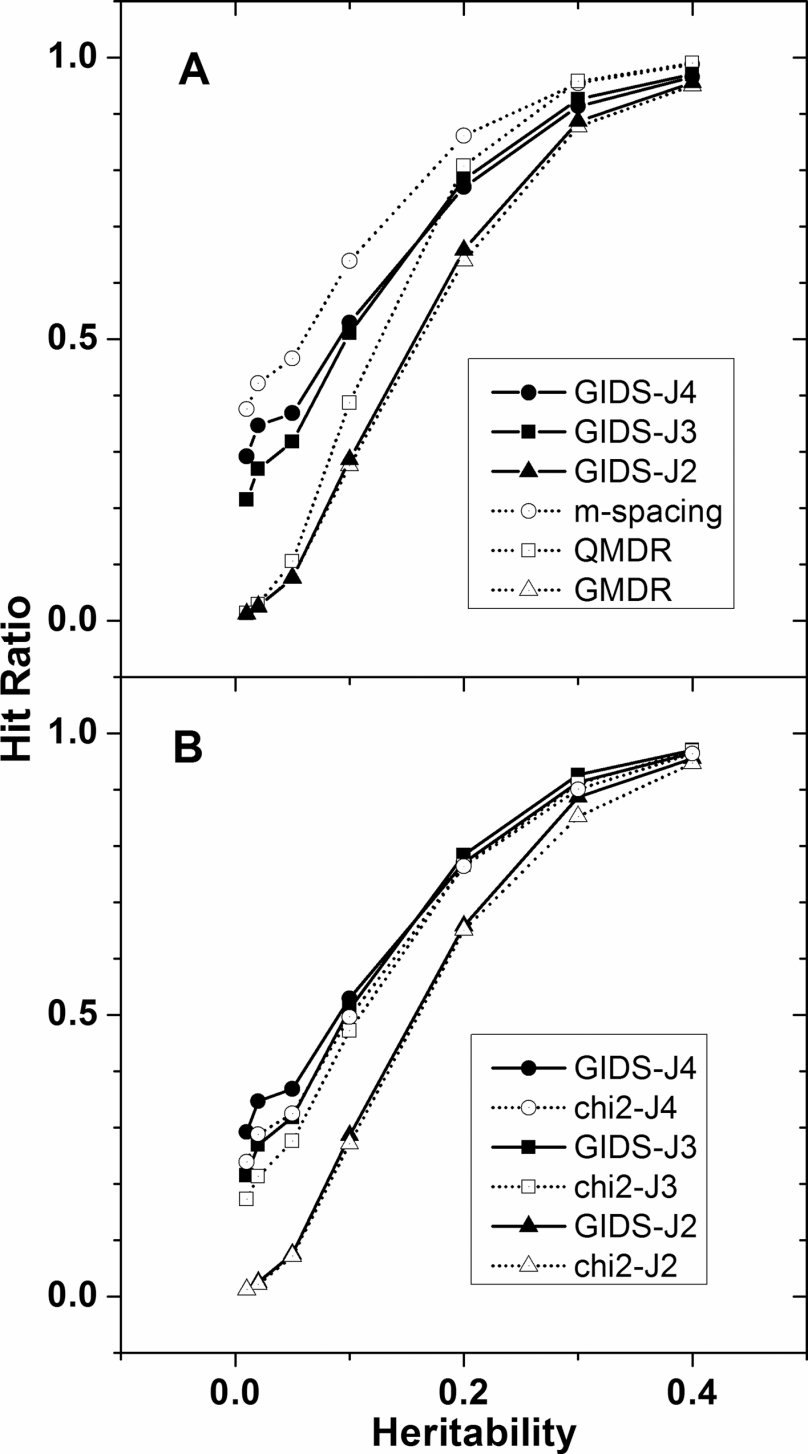
Comparison of performance (simulation dataset II). A simulation dataset whose quantitative phenotype distribution was made out of three Gaussians was analyzed for the 2-order interaction. Hit ratios were obtained using several methods and compared (A) using the same dataset. GIDS-J2, -J3, and -J4 use the phenotypes of two, three, and four classes categorized beforehand. GMDR and QMDR accept the quantitative phenotype but perform a dichotomous categorization of their own. Only m-spacing analyzes the quantitative simulation dataset in its original form. Hit ratios from GIDS and *χ*^2^ are also compared (B).

In Fig 4, the performances on the simulated dataset II are examined by the hit ratios plotted for seven different heritability values. Performance evolution of GIDS as the quantitative trait is classified into an increasing number of phenotype classes, *J*, is shown. The filled triangles, squares, and circles indicate the evolved GIDS with *J* = 2, 3, and 4, respectively, which means that the phenotypes are classified into 2, 3, and 4 categories. Effect of varying number of the trait classes, due to altered classification criteria, on the performance of the GIDS would be demonstrated by comparing the performances of GIDS-J2, -J3, and -J4. Because GIDS is intended for multi-class phenotypes that are intrinsically categorical or classified beforehand from a quantitative trait, it does not provide its own categorization scheme. While the actual classification rules or agreements will vary from phenotype to phenotype, we applied a simple ‘beforehand’ categorization rule such that the quantitative trait distribution would be divided into 2, 3, or 4 partitions with similar areas. Specifically we used the thresholds of (*μ*), (*μ–z0σ*, *μ* + *zσ*), and (μ*—zσ*, μ, μ + *zσ*) for the categorization of *J* = 2, 3, and 4, respectively. Here μ and *σ* represent the overall mean and standard deviation of the distribution where *z* = 0.43, 0.67 for *J* = 3, 4, respectively. A clear distinction can be seen between GIDS-J2 and GIDS-J3, or -J4. Because the dataset was prepared by mixing three Gaussian distributions, *J* = 3 or 4 may be expected to be more appropriately categorized than *J* = 2. A consistently better performance has been observed from GIDS-J3, and -J4 than from GIDS-J2. Recalling that the three datasets analyzed were made from a single original dataset, the performance difference between them may be interpreted as the correct reflection, by GIDS, of the adequacy of the categorization.

In Fig 4A, we compared the performance of GIDS-J2, -J3, and -J4 with the result from the m-spacing method, which does not require trait categorization, and with GMDR and QMDR, which have their own sophisticated categorization methods. The m-spacing method analyzes a quantitative trait. In other words, it is not required to classify the trait distribution [23]. It is shown that m-spacing performs the best as can be expected for a method that may have the least chance of distorting the quantitative trait distribution. GMDR uses its own definition of a score value to replace the ratio of cases to controls [10]. QMDR compares the mean value of each multi-locus genotype to the overall mean and then uses a T-test [11]. Common to both methods is that only one threshold is introduced. Therefore, their classification is intrinsically dichotomous. Now the intended comparison may provide an idea for a better way to analyze a genomic data with a quantitative trait. Treating the quantitative trait in its original form sets the best performance while all three dichotomous methods, including GIDS-J2, are found to be least effective. Categorizing the quantitative trait into three or four classes made GIDS show the performance in between the most and least effective methods, as can be seen by the GIDS-J3, and -J4 lines in Fig 4A. The dataset used was simulated under a particular scheme and may not be generalized, but analyzing arbitrary quantitative traits with dichotomous classification may not be suitable. Existence of a method such as GIDS that is capable of analyzing a multi-class trait may support more flexible categorization of real phenotypes, usually observed in quantitative form, beyond simple case-control binary classification.

Comparison of the performance of GIDS-J2, -J3, and–J4 with the corresponding *χ*^2^ tests is shown in Fig 4B, noting that *χ*^2^ also examines the deviation from the expected distribution similarly to GIDS. GIDS consistently showed better performance than *χ*^2^, regardless of the number of categorization, *J*. Improvements was about 10% with *J* = 3, 4, and about 5% with *J* = 2. Difference in improvement may be due to the difference in the denominator of the equations of GIDS and *χ*^2^. While GIDS has a normalizing denominator that has *J* in it, *χ*^2^ has an indefinite maximal end value that tends to grow as *J* increases. This may also increase the chance of overestimation that could reduce the performance.

### Type I error rate

The type I error rate was estimated using the null dataset generated following the same process as for simulation II, only without the causal pair. All the pairs among the 20 SNPs should not be found to have any noticeable association. The ratio of the permutation p-values smaller than or equal to a significance level, *α*, was taken as the type I error rate. Each data file in the null dataset was permuted by 1000 times to ensure the accuracy of the type I error, in percent, to one decimal place; *α* was set as 0.05. Estimation is reported in Table 1 separately for *J* = 2, 3, and 4 with each of the examined MAFs and heritability values. Maximum and minimum type I error rates in percent were (5.6, 4.9), (5.4, 4.8), and (5.6, 5.0) for *J* = 2, 3, and 4, respectively, resulting in averages of 5.2, 5.2, and 5.3% for the corresponding *J*. Therefore it can be concluded that GIDS preserves the type I error rate over the conditions examined without any dependence on the number of outcome classes set for the analysis.

**Table 1:** Type I error estimation with a significance level (*α*) of 0.05.

Type I error rate (%)		J2	J3	J4
**MAF**	**0.2**	5.3	5.1	5.3
	**0.4**	5.2	5.2	5.3
**Heritability**	**0.01**	4.9	5.4	5.2
	**0.02**	5.6	5.0	5.1
	**0.05**	5.4	4.8	5.0
	**0.1**	5.3	5.1	5.4
	**0.2**	5.0	5.4	5.2
	**0.3**	5.4	5.3	5.6
	**0.4**	5.2	5.3	5.4
**Overall**		**5.2**	**5.2**	**5.3**

### Classification of blood pressure

The Seventh Report of the Joint National Committee on Prevention, Detection, Evaluation, and Treatment of High Blood Pressure (JNC 7) [16] classified blood pressure (BP) states as normal, prehypertension, and hypertension by considering the systolic (SBP) and the diastolic (DBP) blood pressure simultaneously. Because SBP and DBP are separate observables [24], their values are grouped into three sub-ranges themselves, with thresholds of (120, 140) mmHg for SBP and (80, 90) mmHg for DBP. Then nine distinct categories would be made by combining the two observables as tabulated in Table 2. Note that SBP and DBP are not interlocked and are known to have independent relationships to the risk of cardiovascular disease [24]. Thus, examining the genomic association with the three BP classes as in previous work [25–27] may not reflect the detailed cardiovascular states. In other words, nine composite classes may be expected to yield clearer genomic association because they are retaining the individual states that would merge into a single one if three BP classes were used. Because GIDS does not limit the number of phenotypic classes, composite phenotype of SBP and DBP may well be suitable for the application of the method.

**Table 2:** Classification of blood pressure (BP) with simultaneous consideration of systolic (SBP) and diastolic (DBP) blood pressure.

SBP(mmHg)	DBP(mmHg)	Outcome class	BP classification
<120	<80	1	normal
<120	80≤ and <90	2	prehypertension
<120	≥90	3	hypertension
120≤ and <140	<80	4	prehypertension
120≤ and <140	80≤ and <90	5	prehypertension
120≤ and <140	≥90	6	hypertension
≥140	<80	7	hypertension
≥140	80≤ and <90	8	hypertension
≥140	≥90	9	hypertension

### Real data analysis with KARE

**Fig 5:**
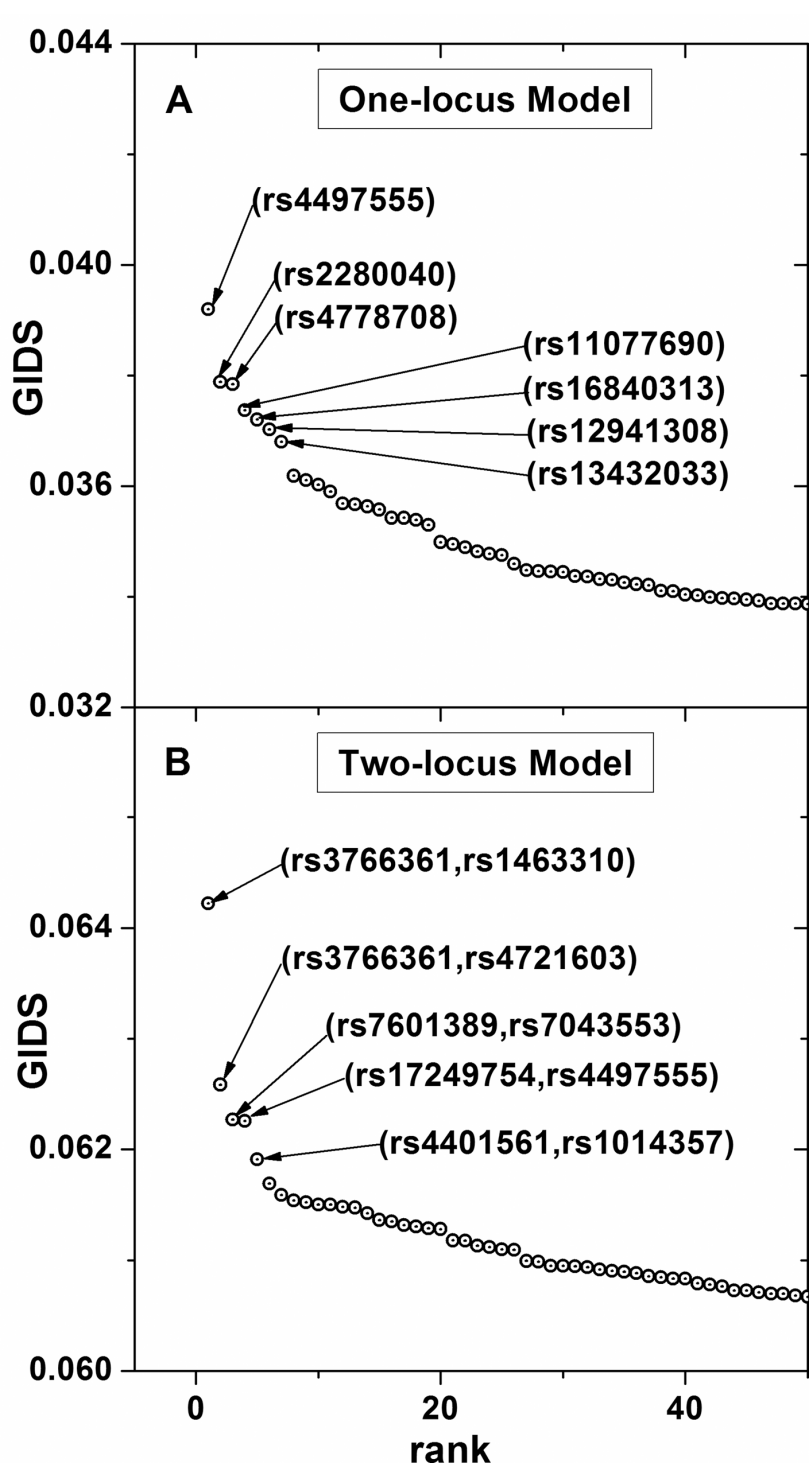
Top associated SNPs and SNP pairs with BP. Applying GIDS to the KARE dataset with the nine composite classes of BP identified the top associated SNPs in the one-locus model (A) and the interacting SNP pairs in the two-locus model (B).

The Korean Association Resource (KARE) project [18] provided an extensive set of various phenotypes from the samples of 8,842 individuals along with 327,872 SNPs spanning over 22 chromosomes. Among them we analyzed the phenotypes of SBP and DBP following the classification by JNC 7. First, SBP and DBP distributions were grouped by three sub-ranges according to the thresholds provided by JNC 7, as described in the classification of BP subsection. Then each sample was categorized into one of the nine classes following the classification scheme in Table 2 so that phenotypes of SBP and DBP from KARE were treated as a single BP phenotype with nine classes. That is, *J* was set as 9 in Eq (2). Then the association strengths were estimated by GIDS for one- and two-locus models. Top association results are shown as a scree plot [28] in Fig 5, where the top associated SNPs in the one-locus model are identified and shown in (A) and the top associated interacting SNP pairs given in (B). A main effect was most prominent with rs4497555 well above the other top SNPs. The top interacting SNP pair, with which GIDS gave the largest value, was (rs3766361, rs1463310). Detailed lists of the most associated SNPs are presented in Tables 3 and 4 for one- and two-locus models, respectively. The significance of each SNP or SNP pair is given by the permutated p-values. Multiple comparisons have been accounted for when estimating p-values by constructing a single common null distribution of GIDS values for each model [19–21]. After each permutation, the maximum GIDS value was sought among all possible SNP or SNP pairs. After all the permutation the collection of those maximum GIDS values formed the null distribution used as the criteria for estimating the p-values. A total of 10,000 permutations were performed. With such estimated p-values, we report single SNPs, as well as interacting SNP pairs, that are newly found to have strong associations with the BP states. Nine BP classes may preserve the individual state of SBP and DBP better than three BP classes. This may have critical importance, because SBP and DBP are independently related to cardiovascular disease [24], such that the association would be better estimated with the categorization of BP reflecting their individual variation as much as possible.

**Table 3:** Top associated SNPs in the one-locus model with permutation p-values.

One-locus Model			
SNP	Chromosome	GIDS	p-value
rs4497555	13	0.0392	0.0024
rs2280040	4	0.0379	0.0062
rs4778708	15	0.0378	0.0063
rs11077690	17	0.0374	0.0095
rs16840313	4	0.0372	0.0113
rs12941308	17	0.0370	0.0126
rs13432033	2	0.0368	0.0149

**Table 4:** Top associated interacting SNP pairs in the two-locus model with permutation p-values.

Two-locus Model				
SNP1	SNP2	Chromosomes	GIDS	p-value
rs3766361	rs1463310	1,7	0.0642	0.0002
rs3766361	rs4721603	1,7	0.0626	0.0003
rs7601389	rs7043553	2,9	0.0623	0.0005
rs17249754	rs4497555	12,13	0.0623	0.0005
rs4401561	rs1014357	5,11	0.0619	0.0006

## Discussion

We propose a method to estimate genomic association and gene-gene interactions even when an outcome classification contains more than two classes. The lack of a mathematical limit on the number of outcome classes analyzable is mainly due to circumventing the explicit use of the concept of risk status. To apply the MDR method, we need to set the threshold for the discrimination of high and low risk. If only one threshold is required, as for binary outcomes, the odds ratio can be used to set a well-defined threshold value. But when considering an outcome with three or more classes, setting the multiple threshold values required would become increasingly ambiguous as the number of classes increases. Therefore, within the scheme of a confusion matrix, there may not be a straightforward way to measure the association between genotypic multifactor classes and multi-class phenotypes. The main advantage of using GIDS is the systematic extension of the number of analyzable outcome classes to arbitrary numbers by making the explicit use of the risk status unnecessary.

Many real phenotype data are observed as continuous variables in their intrinsic forms, which may be classified afterward and analysed as categorical variables. Classification may be strict or more inclusive. For example, for blood pressure, the detailed number of categories was changed from seven to six when the Seventh Report of the Joint National Committee on Prevention, Detection, Evaluation, and Treatment of High Blood Pressure (JNC7) was introduced, replacing JNC6 [16]. Obesity may be classified into two, four, or five categories [15]. Although the final forms of the exemplified phenotypes are categorical, the originally obtained observables are all quantitative and the classification criteria are more or less the agreements that may be altered. Quantitative phenotypes may be analyzed better with a multi-class categorization. GMDR and QMDR can handle a quantitative phenotype value. However, these methods remain as binary categorizations. Here, we suggest GIDS as a way of analyzing a multi-class outcome categorized beforehand, rather than as a categorization scheme. GIDS may be regarded as a generalized BA if one recalls the analytic equivalence between BA and IDS (see the Methods section) and that IDS is a special case of GIDS when *J* = 2.

Another important contribution of GIDS is that multiple phenotypes can be analyzed as if they are a single composite trait. As can be seen in Table 2, the nine composite states of SBP and DBP nominally have three classification categories, although the detailed disease status may be different within the same category. For example, consider two subjects with (SBP, DBP) of (140, 70) mmHg and (115, 90) mmHg, respectively. They are most likely to have different physiological conditions, although both of them are classified as having hypertension. Therefore analysis using only the three categories may not be successful in finding a genetic association for the hypertension. This would make a good challenge for a multi-class phenotype analysis. Association analysis using the GIDS would be intrinsically suitable for multivariate analysis. We can apply the method by arranging the phenotypic outcome classes similarly as in genotypic multifactor classes.

Although we have not used any cross-validation (CV), it can be easily implemented into our procedure. For example, a standard MDR procedure with CV can be proposed by using GIDS. If the best model is chosen based on the value of GIDS itself, then we need to consider overfitting. Instead of using CV, however, we proposed using a permutation approach based on randomization [19]. By ‘randomization’, as is named in [19], a series of procedures has been suggested that leads to the production of a null distribution and guides the test for the null hypothesis by yielding the p-values. P-values are estimated with a null distribution constructed with the collection of the maximum GIDS values obtained from each of the iteration of random permutation of the whole dataset. P-values based on these null distributions are used to choose the best model. For example, one of the data file in simulation I was analyzed to give the top ranked SNP combinations with GIDS and p-values in 1,2,3,4-order interactions as follows. (SNPs; GIDS, p-value) = (7; 0.1433, 0.0090), (1,2; 0.2596, 0.0001), (1,2,5; 0.3230, 0.0007), (1,2,5,11; 0.4567, 0.0009). Then the best model can be identified as (1,2) among the top ranked models from four different orders of interactions.

In summary, the proposed method could be applied in genomic association studies with a multi-class outcome either as a single phenotype or a multivariate composite phenotype.
